# Ginsenoside Rg1 Improves Differentiation by Inhibiting Senescence of Human Bone Marrow Mesenchymal Stem Cell via GSK-3*β* and *β*-Catenin

**DOI:** 10.1155/2020/2365814

**Published:** 2020-05-26

**Authors:** Ziling Wang, Rong Jiang, Lu Wang, Xiongbin Chen, Yue Xiang, Linbo Chen, Minghe Xiao, Li Ling, Yaping Wang

**Affiliations:** ^1^Department of Histology and Embryology, Laboratory of Stem Cells and Tissue Engineering, Chongqing Medical University, Chongqing 400016, China; ^2^Department of Anatomy and Histology and Embryology, Basic Medical College, Chengdu University of Traditional Chinese Medicine, Sichuan 610075, China; ^3^Department of Obstetrics and Gynecology, The Second Affiliated Hospital of Chongqing Medical University, Chongqing 400010, China

## Abstract

**Objectives:**

To demonstrate the effect of Ginsenoside Rg1 on the differentiation of human bone marrow-derived mesenchymal stem cells (hBM-MSCs). Subsequently, a rational mechanism for the detection of Rg1 which affects mesenchymal stem cell differentiation was explored.

**Methods:**

Flow cytometry is used for cell identification. The differentiation ability of hBM-MSCs was studied by differentiation culture. SA-*β*-gal staining is used to detect cell senescence levels. Western blot and immunofluorescence were used to determine protein expression levels. RT-qPCR is used to detect mRNA expression levels.

**Results:**

Rg1 regulates the differentiation of hBM-MSCs. Differentiation culture analysis showed that Rg1 promoted cells to osteogenesis and chondrogenesis. Western blot results showed that Rg1 regulated the overactivation of the *β*-catenin signaling pathway and significantly adjusted the phosphorylation of GSK-3*β*. GSK-3*β* inhibitor (Licl) significantly increased Rg1-induced phosphorylation of GSK-3*β*, which in turn reduced Rg1-induced differentiation of hBM-MSCs.

**Conclusion:**

Ginsenoside Rg1 can reduce the excessive activation of the Wnt pathway in senescent cells by inhibiting the phosphorylation of GSK-3*β* and regulate the mesenchymal stem cell differentiation ability.

## 1. Introduction

Adult stem cell senescence is the latest theory of the occurrence and development of human aging and senile diseases [1–4]. Stem cells can maintain and control the homeostasis of a normal body [5, 6], the aging of stem cells which leads to the degeneration of body structure and function, and the occurrence of related senile diseases. For the prevention and treatment of senile degenerative diseases, it is of great value to study the mechanism and control measures of stem cell aging and to find ways to stimulate stem cell activity. Mesenchymal stem cells (MSCs) are derived from the mesoderm and can proliferate and differentiate into fibroblasts, reticular cells, macrophages and endothelial cells, fat cells, osteoblasts, and hematopoietic stromal cells [7]. Previous studies [8, 9] have proved that with the increase of age, hBM-MSCs will show dynamic aging biological changes and then accompany the occurrence and development of senile diseases.

Panax ginseng is a traditional Chinese medicine to replenish “qi”. According to Shen Nong's Herbal Classic [10], P. ginseng can “tonify organs, reduce the eclampsia, improving eyesight, good for the brain, and remove evil spirits”, and “consistent and correct use of P. ginseng can prolong life”. Modern medical research and laboratory analysis also show [11–13] that ginsenoside Rg1 is an important chemical monomer in P. ginseng, which has obvious effects on regulating people's central nervous system, cardiovascular system, antifatigue, and regulating material metabolism. This research group has long been committed in combining the traditional Chinese medicine concept and stem cell theory and striving to find a way to delay the aging of the body through the collision of the traditional “qi and blood” theory and the stem cell theory, so as to make the body “healthy when old” and avoid the “premature aging of the body”. Previous studies [14–17] have shown that ginsenoside Rg1 can antagonize the oxidative damage of the body and regulate the aging of neural stem cells and hematopoietic stem cells by affecting the oxidative stress mechanism of cells. So, can ginsenoside Rg1 regulate the aging of human bone marrow mesenchymal stem cells? What is the possible mechanism?

The Wnt/*β*-catenin signaling pathway is an important stem-cell related pathway [18]. The Wnt pathway is conserved during evolution and has a wide range of biological functions during development [19–21]. It has been reported [22–25] that the activation level of the *β*-catenin signaling pathway is closely related to the level of stem cell senescence. In our previous studies [26–28], we found that stem cell senescence is due to changes in the living environment of stem cells and is due to changes in the signaling pathways associated with changes in stem cell microenvironment. Among them, the changes of the *β*-catenin signaling pathway have a wide and important relationship with changes in stem cell function. Recent studies [24, 29, 30] have shown that the *β*-catenin signaling pathway has a wide range of biological effects on hematopoietic stem cell senescence, and hematopoietic stem cell senescence can be produced by overactivation of the *β*-catenin signaling pathway. In contrast, inhibition of the *β*-catenin signaling pathway can attenuate the senescence of hematopoietic stem cells. So, is aging of human bone marrow mesenchymal stem cells (hBM-MSCs) associated with the *β*-catenin signaling pathway?

In the current study, the effect of Rg1 on the differentiation of hBM-MSCs was determined, and the roles of the Wnt/*β*-catenin signaling pathway were studied. These findings may support the use of Rg1 in promoting stem cell viability and differentiation, as well as in tissue engineering and clinical therapy.

## 2. Materials and Methods

### 2.1. Isolation and Culture of hBM-MSCs

Human bone marrow-derived mesenchymal stem cells were isolated from the bone marrow. The bone marrow from healthy donors aged 18 to 80 (*n* > 20/group) was collected from volunteers who received bone marrow puncture at the First Affiliated Hospital of Chongqing Medical University, China. The study was approved by the ethics committee of Chongqing medical university.

The bone marrow cells were mixed with the red blood cell cracking liquid and the sedimentation (volume 5 : 1) at 4°C for 5 min. Mononuclear cells were separated from the residue by using an isolated lymphocyte separation medium. The isolated hBM-MSCs were resuspended in DMEM/F12 supplemented with 10% FBS, 1% penicillin, and 1% streptomycin. Cells were cultured at a density of 5 × 10^5^/cm^2^ in a humidified environment at 37°C with 5% CO_2_. After about 20 to 25 d, the cells were cultured and fused at 90 to 100 percent, followed by subculture. Passage cells were carried every 7-10 d. The third and sixth sections of hBM-MSCs (p3-p6) were used for the experiment. The growth and morphology of the cells were observed with an inverted microscope (Olympus Corporation, Tokyo, Japan).

### 2.2. Flow Cytometry

Flow cytometry was used to detect the expression of hBM-MSC surface antigen markers. The cells (>1 × 10^6^ in each group) of each group were suspended in PBS containing 2% BSA, fluorescein isothiocyanate- (FITC-) labeled or phycoerythrin- (PE-) labeled specific antibodies FITC-CD105, FITC-CD45, FITC-CD34, FITC-CD19, FITC-CD14, FITC HLA-DR, FITC-CD90, PE-CD73, PE-CD11b which were incubated in accordance with the specification at 4°C for 30 min in dark. The results were used Cell Quest software for data processing.

### 2.3. Optimization of Rg1 Treatment Protocol and Dosage

The Rg1 was purchased from Jilin Hongjiu Biotechnology Co. Ltd. Rg1 is white solid powder, slightly soluble in water, and soluble in DMSO. Thus, Rg1 is configured with a high concentration of DMSO solution. Cells in the Rg1 group were exposed to Rg1, and cells in the control group received pseudotreatment (without Rg1). The optimal concentration and time of the drug were determined by a CCK-8 method and cell proliferation analysis.

### 2.4. EdU Assay

HBM-MSCs were inoculated in 24-well plates at a concentration of 1.5 × 105/mL and cultured in Edu solution for 24 h. 4% paraformaldehyde was fixed 30 min at room temperature. Washed cells were permeabilized 0.5% Triton X-100 for 20 min. Then, the cells were incubated with 1X Apollo® reaction cocktail for 30 min and stained using 1X Hoechst33342 for 30 min and imaged under a fluorescent microscope (Olympus Corporation). The proliferation rate was defined as the ratio of Edu-positive cells (labeled red fluorescence) to hoechst33342-positive cells (labeled blue fluorescence).

### 2.5. Senescence-Associated *β*-Galactosidase (SA-*β*-Gal) Staining

The SA-*β*-gal staining was performed according to the manufacturer's instructions. Cells were prepared as previously described, fix in 4% paraformaldehyde for 5 minutes, washed twice with PBS, and plastic wrap sealed in a dyeing box, stained at 37°C overnight. The cytoplasm stained blue is senescent cells. Collection of images was done by an inverted microscope and optical density analysis by Image-Pro Plus 6.0 software.

### 2.6. In Vitro Differentiation

#### 2.6.1. Osteogenesis

When the cell adherence rate reached 50%-70%, the conventional medium was replaced with ODM, and the culture was replaced with ODM every 3 d for 21 d. Rg1 was added to ODM media in the Rg1 treatment group for 21 days. After 21 d, it was soaked in 4% paraformaldehyde for 30 min and washed with PBS for 3 times. Then, 1% alizarin red S (pH = 4.2) was used for 5 minutes.

#### 2.6.2. Adipogenesis

When cell adherent reached 100%, use ADM instead of conventional culture medium, replace ADM culture every 3 d, and cultivate for 21 d. Rg1 was added to ADM media in the Rg1 treatment group for 21 days. After 21 d, the cells were washed with 4% paraformaldehyde for 30 min and washed with PBS for 3 times. Then, dye with oil red O for 60 minutes.

#### 2.6.3. Chondrogenesis

When cell adherent reached 70%-80%, use CDM instead of conventional culture medium, replace CDM culture every 3 d, and cultivate for 21 d. Rg1 was added to CDM media in the Rg1 treatment group for 21 days. After 21 d, the cells were washed with 4% paraformaldehyde for 30 min and washed with PBS for 3 times. Then, dye with Alcian blue for 30 minutes.

Differentiation kits and staining solution were purchased from Cyagen Biosciences Inc. (Suzhou, China). Images were observed and collected using an inverted microscope (Olympus Corporation).

### 2.7. Immunofluorescence Staining

Cells prepared as previously described were fixed by 4% paraformaldehyde for 20 mins. After washing with 0.5% Triton X-100 for 20 min, blocking with 10% goat serum was done for 30 mins at room temperature. Then, the cells were incubated with antibodies against *β*-catenin (1 : 100) overnight at 4°C. In the next day, the cells were warmed at room temperature for 30 mins and washed three times. Then, the cy3-labeled secondary antibody (1 : 300) was incubated at 37°C for 1 h. DAPI is used for nuclear staining. All slides were observed directly under a fluorescence microscope (LSM510; Carl Zeiss, Jena, Germany). Antibodies were purchased from Cell Signaling Technology Inc. (Boston, MA, USA).

### 2.8. Western Blot

Total protein was extracted by radioimmunoprecipitation, the lysate buffer was mixed with a 1% protease inhibitor cocktail (Beyotime Institute of Biotechnology), and the concentration was determined by the bicinchoninic acid method. Samples containing 40 g proteins were separated by 12% SDS PAGE and transferred to polyvinylidene fluoride membrane. At room temperature, it was sealed with skim milk powder (5% dissolved in TBS tween 20) for 2 h, incubated overnight in 4°C (1 : 1000 diluted in primary antibody dilution buffer) with specific primary antibodies for *β*-catenin, GSK-3*β*, *β*-actin (all from Cell Signaling Technology, Boston, MA, USA), LEF, TCF-4, and C-myc (all from Proteintech Group, Inc., China). Incubate at room temperature for 90 minutes with a secondary antibody (1 : 10000 TBS-tween). Use an enhanced chemiluminescence detection system (Pierce; Thermo Fisher Scientific, Inc., Waltham, MA, USA), with *β*-actin (1 : 1,000 diluted antibody dilution buffer) as internal control. The integrated optical density was quantified by Image Lab 5.2.1 (Bio-Rad Laboratories, Inc., Hercules, CA, USA).

### 2.9. Real-Time Quantitative RT-PCR

Total RNA was isolated with trizol reagent (Invitrogen. USA) according to the manufacturer's agreement. The first strand of cDNA was constructed by TaqMan RT reagent (Applied Biosystems, USA). SYBR green Supermix (Bio-Rad) was used to perform real-time quantitative PCR on the circulator real-time detection system (Bio-Rad). The mRNA expression level and GAPDH were normalized, and the comparative cycle threshold method was used for analysis. The mean ± standard deviation of three independent experiments was measured. The PCR primers used are provided in the supporting information (Tables 1 and 2).

### 2.10. Statistical Analysis

All data were expressed as mean ± standard deviation. All statistical analyses were performed using one-way anova, and Fisher's least significant difference test was performed using SPSS v20.0 (IBM Corp., Armonk, NY, USA). *P* < 0.05 was considered statistically significant.

## 3. Results

### 3.1. Conventional Bone Biopsy and Morphometric Determination

**Figure 1:**
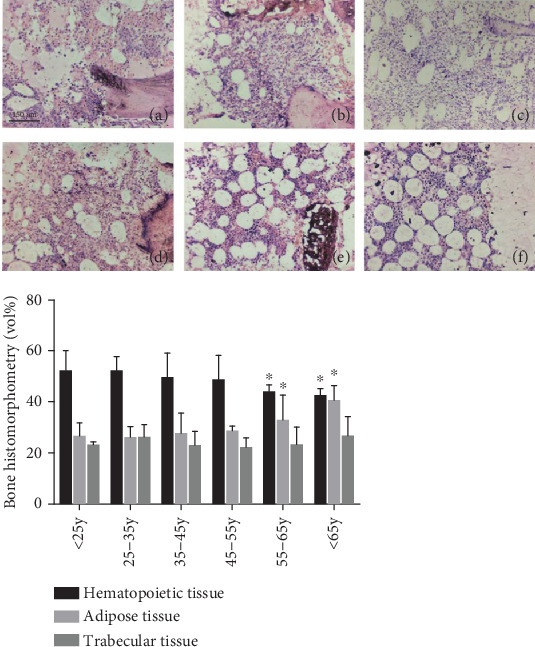
Conventional bone biopsy and morphometry. Bone marrow biopsy specimens from patients of different ages (*n* > 20/group) were collected for Wright staining. The amount of each tissue was checked using a morphometric software. The histogram shows the results of the morphometric measurements. (a) <25 years old group; (b) 25–35 years old group; (c) 35–45 years old group; (d) 45–55 years old group; (e) 55–65 years old group; and (f) >65 years old group.

Bone marrow biopsy is a way to understand the bone marrow. However, the biopsy is more detailed and comprehensive than the puncture on the bone marrow status. For certain diseases, such as myelofibrosis, bone marrow punctures often fail due to “dry pumping”, and bone marrow biopsy is often successful. We clinically collected patient biopsy specimens for Wright's staining. The content of each tissue was examined using morphometric software. The results (Figure 1) showed that with the increase of age, the proportion of hematopoietic tissue and trabecular bone tissue in bone marrow tissue decreased, and the proportion of adipose tissue increased.

### 3.2. The Identifying of hBM-MSCs

**Figure 2:**
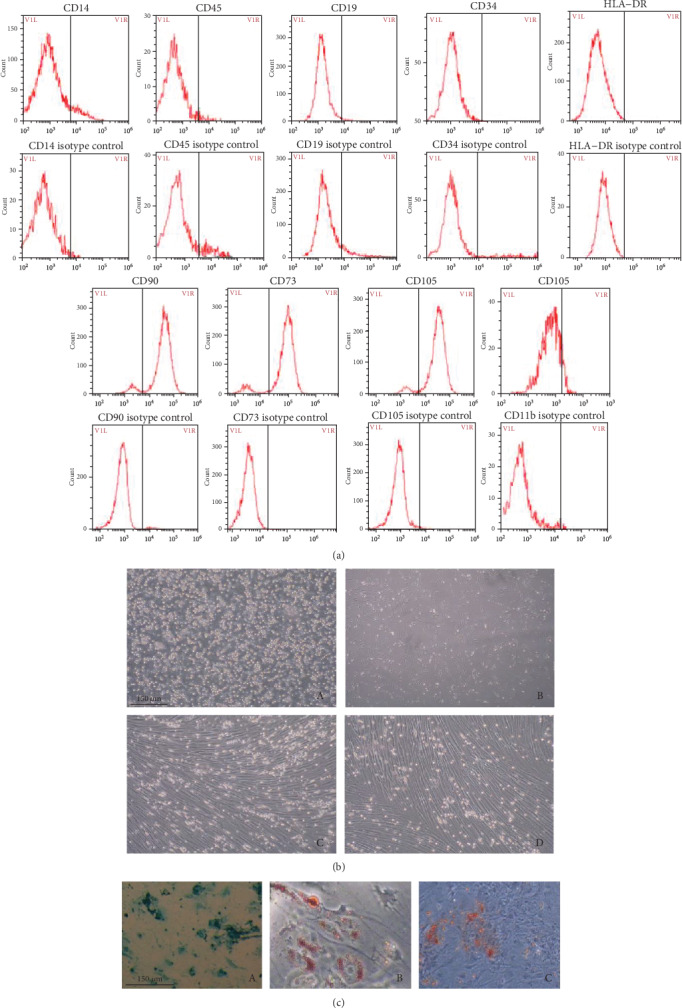
Identification of human bone marrow mesenchymal stem cells. Cells were identified by the surface markers, cell morphology, and differentiation ability. (a) The cells were stained with fluorescein isothiocyanate- (FITC-) or PE-conjugated antibodies or with immunoglobulin isotype control antibodies. (b) The morphology was observed using phase-contrast microscopy at different phases (24 h (b-A), 3 d (b-B), 14 d (b-C), and subcultured hBM-MSCs (P3) (b-D), 100x). (c) The differentiation of hBM-MSCs, using induction medium inducing cells to chondrogenesis (c-A), lipoblast (c-B), and osteogenesis (c-C). Afterward, the special staining was used to detect the differentiation.

The cell morphology, the potential of multipotential differentiation, and the surface markers would be the key to identify the stem cells. Accordingly, experiments were under ways to ensure the validity of cells. The result of cell culture shows that the cells are generally elongated and spindle-shaped and its growth is characteristics of screw growth (Figure 2(b)). Adipogenic differentiation medium-induced hBMSC adipogenesis for 21 d, the result, is shown in Figure 2(c). Chondrogenesis induction on day 21, the Alcan blue staining of hBMSCs, is shown in Figure 2(c). In Figure 2(a), here, is the flow cytometry pattern of the cell phenotype character of hBMSCs, which showed the cells are CD73+, CD90+, CD45-, CD34-, CD14-, and HLA-DR-. All three analyses of cells identified showed these cells which had been purified and cultured are human mesenchymal stem cells [23, 31].

### 3.3. Determination of the Optimal Dose and Exposure Time of Rg1

**Figure 3:**
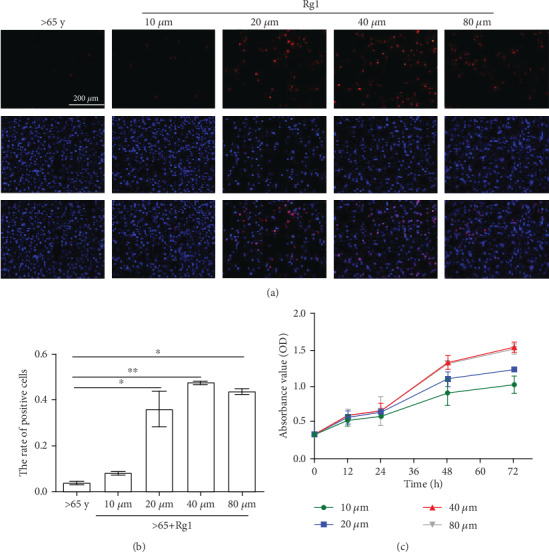
Effects of Rg1 on the growth of human bone marrow-derived mesenchymal stem cells (hBM-MSCs). The Rg1 group was treated with Rg1 in different concentrations and times, respectively. (a, b) Cell proliferation after treatment of senescent mesenchymal stem cells with different concentrations of Rg1. This test was repeated three times. Representative images were shown. (c) The growth curves of hBM-MSCs were measured by Cell Counting Kit-8 (CCK-8) assay. ∗*P* < 0.05, ∗∗*P* < 0.01.

In order to screen the optimal dose and exposure time of Rg1 for hBM-MSCs, we conducted CCK-8 experiment. In general, the mean OD value of the Rg1 group was higher than that of the control group (>65 years old group) (Figure 3). The cell viability increased significantly in the Rg1 groups treated for 24 h (*P* < 0.05) as the Rg1 concentration increased. However, there were no significant differences between the obviously increased groups (*P* > .05). At 48 h, cells treated with 40 *μ*M Rg1 had a significantly higher viability than that of the other group, while those treated with higher concentrations were not changed substantially.

Subsequently, hBM-MSC diffusion was studied by Edu in different groups, and the results showed that the proliferation of hBM-MSCs increased significantly when treated with Rg1 in 40 *μ*M at 48 h.

### 3.4. Effects of Rg1 on Cell Morphology and Senescence

**Figure 4:**
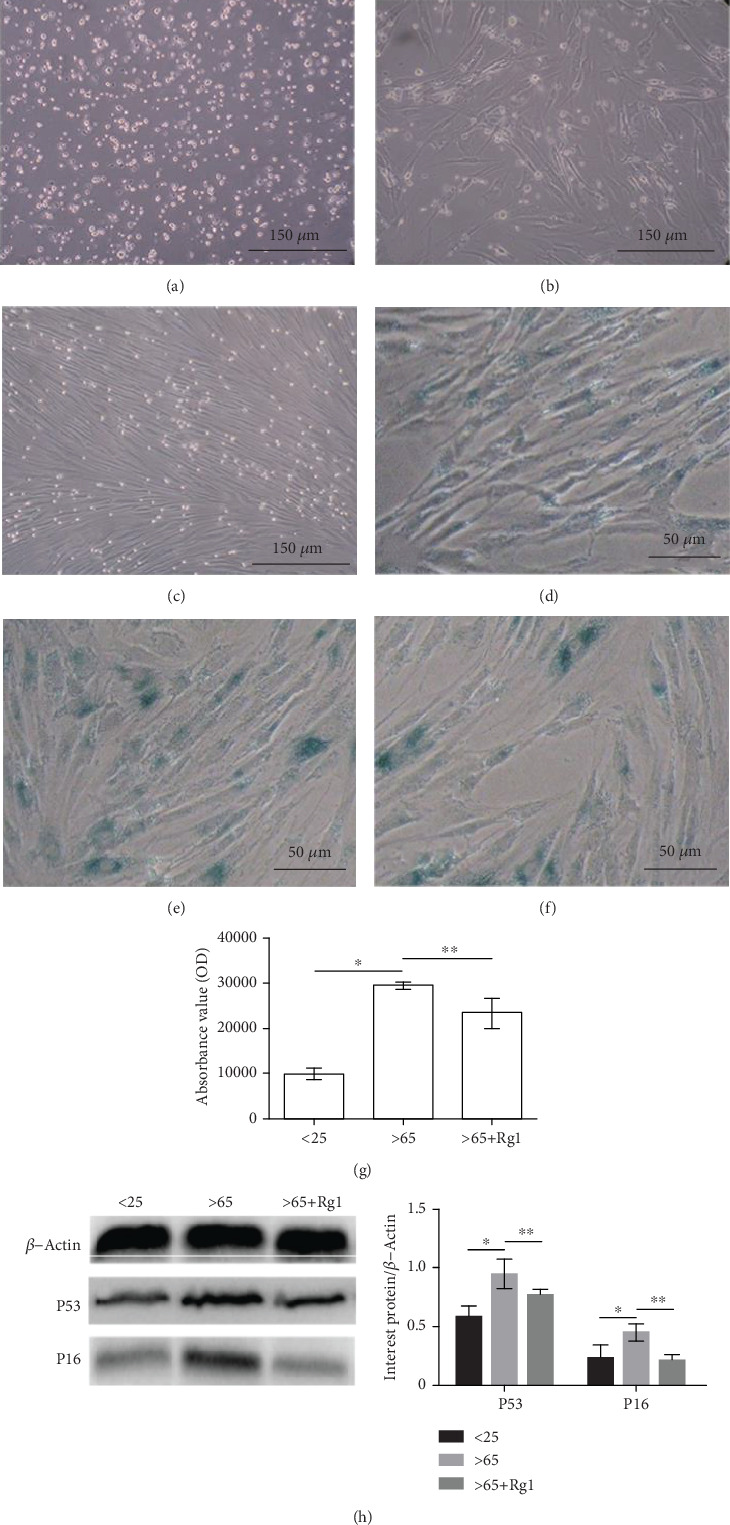
Effect of Rg1 on the senescence and morphology of hBM-MSCs. The cell morphology was observed at (a) 24 h and (b) 72 h and (c) cultured cells to the third generation after isolation. SA-*β*-gal was used to mark the aging cells the cytoplasm of positive cells was stained blue, (d) <25 years old, (e) >65 years old, and (f) >65+Rg1. This test was repeated three times. Representative images were shown. The ROD value was used to evaluate the intensity of SA-*β*-gal-positive staining (g). Western blot was used to detect the protein expression of P53 and P16 (h). *β*-Actin was used as an internal control. ∗∗*P* < .05 vs. >65-year-old group, ∗*P* < .05 vs. < 25-year-old group. SA-*β*-gal: senescence-associated *β*-galactosidase; ROD: relative optical density.

The bone marrow cultured an average of 4 ± 1.18 × 10^7^ per group. After 72 h, 60%-70% of hBM-MSCs were observed to adhere to the culture dish, and some spindle cells were observed (Figure 4(b)). 10-12 d later, hBM-MSCs were 90% to 100% fused. The cells in passage culture also retained the shape of fibroblasts (Figure 4(c)). There was no significant change in cell morphology after treatment with Rg1.

The aging mesenchymal stem cells were dyed blue by using SA-*β*-gal staining [32] (Figures 4(d)–4(f)). The results showed that the positive rate of SA-*β*-Gal staining of hBM-MSCs increased significantly in the aging group (Figure 4(e)). With the treatment of Rg1, the positive rate of SA-*β*-Gal staining of hBM-MSCs decreased (Figure 4(f)). This result indicates that Rg1 can retard the aging of hBM-MSCs. The expression of P53 and P16 protein will increase during aging [33]. The results (Figure 4(h)) showed that Rg1 can decrease the expression of P53 and P16 protein.

### 3.5. Effects of Rg1 on Cell Differentiation Ability of hBM-MSCs

**Figure 5:**
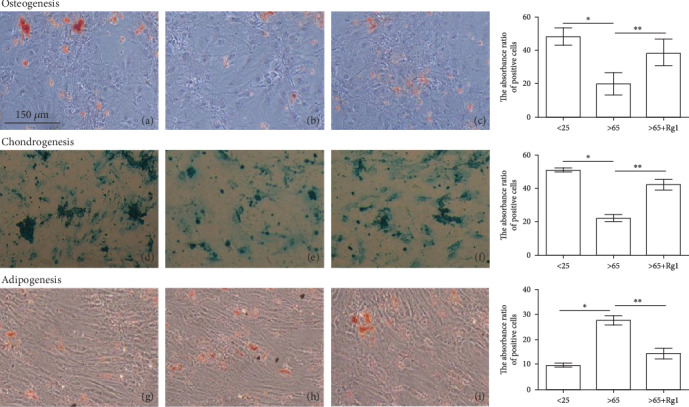
Differentiation potential of human bone marrow-derived mesenchymal stem cells (hBM-MSCs). After 21 d of culture in osteogenic (a–c), or adipogenic (d–f), or chondrogenic (g–i) differentiation medium, the osteogenic, adipogenic, or chondrogenic differentiation of hBM-MSCs was assessed by specific staining. This test was repeated three times. Representative images were shown. Images were taken at 100x magnification. (a, d, and g) the <25-year-old group, (b, e, and h) the >65-year-old group, and (c, f, i) the >65-year-old+Rg1 group.

With mesenchymal stem cells under certain conditions, it can be divided into a variety of functional cells. Studies [34–38] have reported that mesenchymal stem cells have the ability to differentiate into osteocytes, chondrocytes, and adipocytes. The strength of this ability is related to the “ability” of stem cells. The results showed that after osteogenic induction culture, Rg1 treatment of >65-year-old mesenchymal stem cells increased dark red nodules after alizarin red dye staining (Figure 5 (osteogenesis)), the fat-induced culture, Rg1 treatment of >65-year-old mesenchymal stem cells, and oil red O stained with red lipid material reduced (Figure 5 (fat formation)). In cartilage-induced culture, Alcian blue-stained blue cartilage tissue increased after Rg1 treatment of >65-year-old mesenchymal stem cells (Figure 5 (cartilage)). The above results indicate that Rg1 can increase the osteogenic and chondrogenesis differentiation ability of mesenchymal stem cells in the >65-years-old group, which can reduce the adipogenic differentiation ability.

### 3.6. Rg1 Reduces Wnt/*β*-Catenin Pathway Activation in Senile Mesenchymal Stem Cells

**Figure 6:**
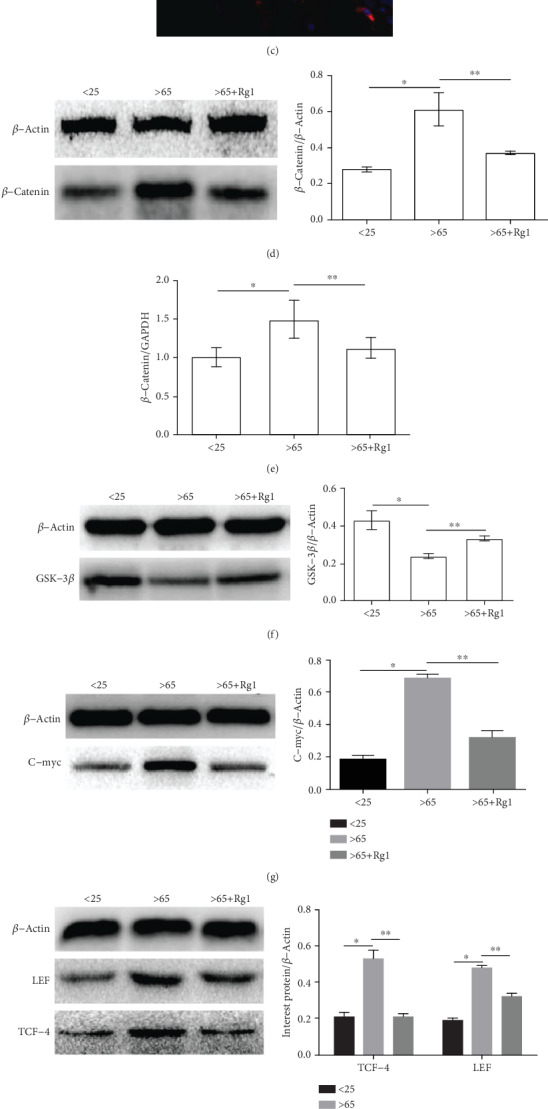
Effect of Rg1 on the expression of Wnt/*β*-catenin signaling pathway-related proteins and mRNA in hBM-MSCs. (a–c) By immunofluorescence, PI (red) cells localize *β*-catenin, DAPI (blue) to visualize the nucleus. (a) the <25-year-old group, (b) the >65-year-old group, and (c) the >65-year-old+Rg1 group. This test was repeated three times. Representative images were shown. (d) Analysis of protein expression of *β*-catenin by Western blot. *β*-Actin was used as an internal control. (e, i) Analysis of mRNA of signaling pathway-related mRNA by RT-PCR; mRNA expression of GAPDH was used as an internal control. (f) Expression of GSK-3*β* protein was analyzed by Western blot. (g, h) Relative protein expression of LEF/TCF and C-myc proteins in different groups, and *β*-Actin was used as an internal control. ∗∗*P* < .05 vs. the >65-year-old group, ∗*P* < .05 vs. the <25-year-old group.

As a multifunctional protein, *β*-catenin is widely distributed in various types of cells [39–41] and plays an important role in cell differentiation [39, 42, 43]. Western blot and immunofluorescence were performed to monitor the key proteins of the Wnt/*β*-catenin signaling pathway. It was found that Rg1 (40 *μ*M, 48 h) significantly reduced *β*-catenin, C-myc, LEF, and TCF-4 levels after treatment of senescent mesenchymal stem cells (Figures 6(d), 6(e), 6(g), and 6(h)). GSK-3*β* levels increased significantly (Figure 6(f)). The results showed that Rg1 reduced the activation of the *β*-catenin pathway in aging hBM-MSCs.

### 3.7. Rg1 Regulates hBM-MSC Differentiation and Is Associated with the Wnt/*β*-Catenin Pathway

To determine whether the effect of Rg1 on the differentiation of aging hBM-MSCs depends on Wnt/*β*-catenin signaling pathways, hBM-MSCs were pretreated with pathway activator Licl (20 mM). The levels of *β*-catenin increased significantly and GSK-3*β* decreased significantly after pretreatment with Licl, indicating that Licl effectively activates the Wnt/*β*-catenin pathway in hBM-MSCs.

**Figure 7:**
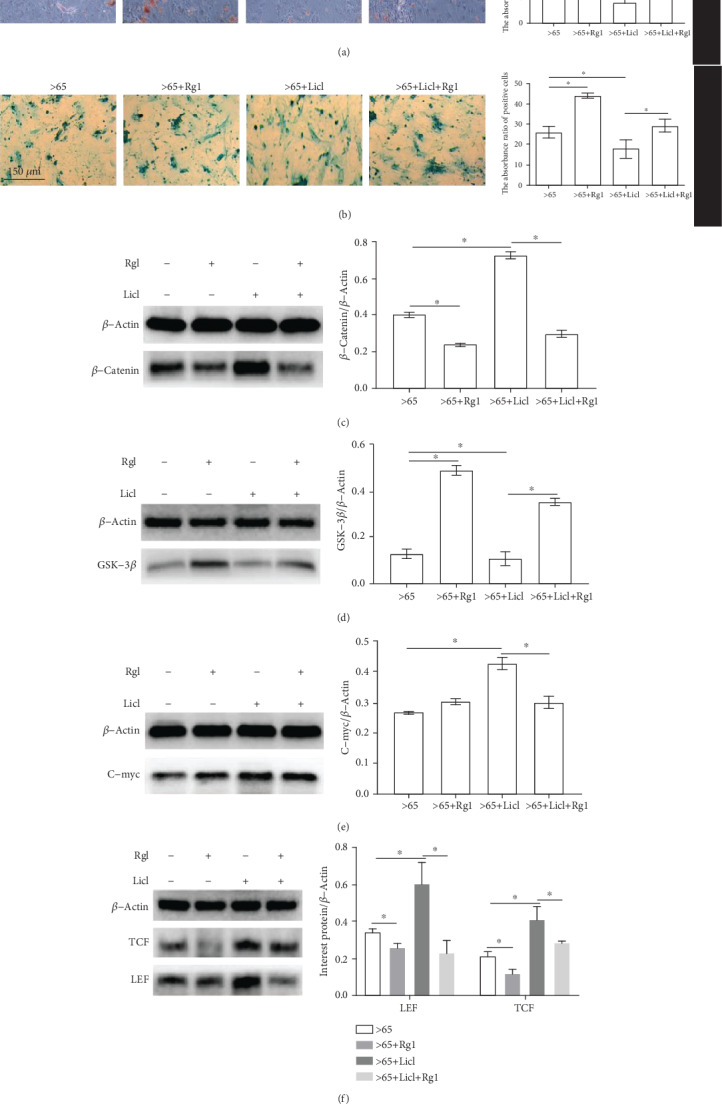
Rg1 regulates differentiation of senile mesenchymal stem cells (hBM-MSCs) related to GSK-3*β* Protein. Cell differentiation capacity was detected by differentiation culture assay in the presence or absence of Licl (a, b). This test was repeated three times. Representative images were shown. *β*-catenin, GSK-3*β*, C-myc, TCF, and LEF were analyzed by Western blot (c–f). ∗*P* < .05.

Cell differentiation was assessed by an induction differentiation kit. The ability of osteoblast and chondrogenic differentiation in the Rg1-treated group was significantly higher than that in the Licl- (Rg1+activator) treated group and the control group (Figures 7(a) and 7(b)). The results showed that after Licl pretreatment, the expression of *β*-catenin, TCF, LEF, and C-myc increased, while the expression of GSK-3*β* decreased. After Rg1 treatment, *β*-catenin protein, TCF, LEF, and C-myc decreased, while GSK-3*β* expression increased (Figures 7(c)–7(f)).

## 4. Discussion

Myelofibrosis (MF) is referred to as myelin. It is a kind of myeloproliferative disease caused by collagen hyperplasia in bone marrow hematopoietic tissue, and its fibrous tissue seriously affects hematopoietic function. The age of onset is between 50 and 70 years old. Some scholars believe that myelofibrosis is caused by abnormal stimulation of hematopoietic stem cells, leading to fibrous tissue hyperplasia, even new bone formation, and bone marrow hematopoietic tissue involvement eventually leading to hematopoietic failure.

During the bone marrow puncture process, it was found that the older the patient, the greater the probability of “dry pumping”. Statistics on bone marrow biopsy results show that as the age increases, the proportion of hematopoietic tissue and trabecular bone in human bone marrow decreases, while the proportion of adipose tissue increases.

In this study, hBM-MSCs were successfully isolated from the human bone marrow. The cultured hBM-MSCs displayed fibroblastic morphology. Previous experiments [44] have shown that the surface markers were similar to those of mesenchymal stem cells, expressing typical mesenchymal markers (CD73, CD90). The expression of cell surface antigens was consistent with the previously reported data of hBM-MSCs. hBM-MSCs can differentiate into osteoblasts, adipocytes, and chondroblasts in vitro. These results indicated that the isolated cells were identified as hBM-MSCs with the common characteristics of pluripotent stem cells.

Mesenchymal stem cells (MSC) are a kind of pluripotent stem cells belonging to the mesoderm [45]. The differentiation direction of mesenchymal stem cells is very important [45–50]. Therefore, it is of great significance to study methods to regulate the differentiation of mesenchymal stem cells. Traditional Chinese medicine is the inheritance of thousands of years of Chinese culture and civilization. It is regarded as a life science with Chinese characteristics. The theory of Traditional Chinese Medicine [24] holds that the stem cells belong to the “ fundamental qi” of the body. Specifically, embryonic stem cells belong to “innate qi”, while adult stem cells belong to “acquired qi”. The situation of “qi” is linked to overall physical fitness. Ginsenoside is an essential medicine for “invigorating qi” in the clinical practice of traditional Chinese medicine. As the main active ingredient of P. ginseng, Rg1 plays an important role in the treatment or adjuvant treatment of various diseases [51–54]. Can stem cell function be activated by replenishing “qi”? Can delay the aging of the body through make stem cell “qi” enough? All of these questions are worthy of in-depth discussion.

Our study found that Rg1-inhibited Wnt/*β*-catenin signaling pathway regulated the differentiation of hBM-MSCs. The Wnt/*β*-catenin signaling pathway is a canonical pathway to regulate stem cell functions by regulating the localization of *β*-catenin, thus modulating the expression of downstream proteins and genes [19, 20, 55]. GSK-3*β* is an important part of the complex degrading *β*-catenin protein in the Wnt/*β*-catenin signal pathway [55]. In the absence of Wnt signaling, *β*-catenin forms polyprotein complexes with Axin, APC (adenomatous polyposis coli), GSK3*β*, and CK1*α*. Then, *β*-catenin is phosphorylated by the kinase in this complex and degraded by E3 ubiquitin ligase. TCF-4 and LEF are important binding sites of *β*-catenin protein after entering the nucleus. C-myc and Cyclin D1 genes are important target genes of the Wnt/*β*-catenin signal pathway. Overactivation of the Wnt/*β*-catenin signal pathway tends *β*-catenin protein to cluster together to enter into the nucleus. Overactivation of the Wnt/*β*-catenin signaling pathway increases the production of *β*-catenin by degrading the complex of APC and GSK-3*β*. As the level of *β*-catenin in the cytoplasm increased, it will transfer to the nucleus and form a complex with family members such as transcription factor TCF/LEF [56–58]. By regulating the expression of target genes such as C-myc and cyclin D1, the physiological function of stem cells is regulated [20].

In this study, the mechanism of Rg1 delaying the senescence of mesenchymal stem cells was further investigated. As an inhibitor of GSK-3*β*, Licl can activate the Wnt/*β*-catenin signaling pathway and aggravate the senescence of mesenchymal stem cells. When ginsenoside Rg1 was added to the Licl group, it was seen that the ability of Rg1+Licl to form bone nodules and cartilage nodules was enhanced compared to the Licl group. It is shown that Rg1 can regulate the decrease of Licl-induced differentiation ability. Western blot showed that expression of *β*-catenin, TCF, C-myc, and LEF in the Rg1+Licl group decreased, and the expression of GSK-3*β* increased. These results indicate that ginsenoside Rg1 can interfere with the activation of the Wnt/*β*-catenin signaling pathway and delay the senescence of hBM-MSCs.

However, the exact mechanism by which Rg1 regulates the Wnt/*β*-catenin signaling pathway remains unclear. It has been reported [59] that Rg1 can antagonize oxidative stress injury and reduce the release of various oxidation factors in cells, including malonaldehyde (MDA) and reactive oxygen species (ROS). Previous studies [60, 61] have shown that Rg1 also have an anti-inflammatory effect in both cells and mice and reduce the expression and release of various inflammatory factor in cells, including interleukin-1 (IL-1), interleukin-6 (IL-6), and tumor necrosis factor-*α* (TNF-*α*). Zhou et al. [62] found the generation of ROS and activation of NF-*κ*B transcription factor, which leads to induction of Wnt ligands such as Wnt1 and Wnt7a and activation of *β*-catenin. The finding showed that oxidative stress is an upstream regulator of Wnt/*β*-catenin, a developmental signal usually silenced in normal adult kidney. Bi et al. [63] found that Rg1 can downregulate cytokine-cytokine receptor interaction, including RELT, TNFRSF8, TNFRSF6B, and EDA2R, which were able to bind tumor necrosis factor receptor-associated factor 1 or combine with Fas ligand TNFSF6 to induce cell death in cells that express this receptor molecule. *β*-Catenin and GSK-3*β* play a regulatory role in these processes [63]. Thus, we speculate that two possible mechanisms might be in charge of the Rg1-regulated Wnt signaling pathway in this study. One is that Rg1 may reduce the expression and release of oxidation factors that cause the regulation of the Wnt signaling pathway. The other is that the gene downgrading by Rg1 can regulate phosphorylation of GSK-3*β* and *β*-catenin. However, this speculation needs to be further studied.

In conclusion, Rg1 can regulate the differentiation of hBM-MSCs, and the Wnt/*β*-catenin signaling pathway may be involved in this process. The findings of this study provide important experimental evidence for the application of Rg1 in the adjuvant therapy of MSCs.

## Figures and Tables

**Table 1:** 

SYBR® premix Taq™II (2x)	5 *μ*L
PCR forward primer (10 *μ*Μ)	0.1 *μ*L
PCR reverse primer (10 *μ*Μ)	0.1 *μ*L
cDNA	2 *μ*L
RNase-free dH_2_O	2.8 *μ*L
Total	10 *μ*L

Reaction conditions: 95°C 30 s, 95°C 5 s, 60°C 30s, circle 40 times.

**Table 2:** Primers used in real-time quantitative PCR.

*β*-Catenin	Forward	5′-AAGCCACAAGATTACAAGAAACGG-3′
	Reverse	5′-CCAAGATCAGCAGTCTCATTCCAA-3′

LEF	Forward	5′-GAAATCATCCCAGCCAGCAA-3′
	Reverse	5′-GGACCCATTTGACATGTACGG-3′

C-myc	Forward	5′-GAGACAGATCAGCAACAACCGA-3′
	Reverse	5′-CTGCTTGGACGGACAGGATG-3′

TCF-4	Forward	5′-GGAAAGAAGAAGAGGCGGTCA-3′
	Reverse	5′-GCACTGTCATCGGAAGGAACG-3′

GAPDH	Forward	5′-GCTACAGCTTCACCACCACAG-3′
	Reverse	5′-GGTCTTTACGGATGTCAACGTC-3′

## Data Availability

Data sharing is not applicable to this article as no new data were created or analyzed in this study.
